# Comparison of perioperative blood loss of three different posterior nonfusion decompression operations for single-segment lumbar disc herniation

**DOI:** 10.3389/fneur.2025.1551742

**Published:** 2025-06-18

**Authors:** Liren Wang, Xing Guo, Zhenjie Song, Hanwu Tang, Haiwei Guo, Ying Li

**Affiliations:** ^1^The Third Affiliated Hospital of Guangzhou University of Chinese Medicine, Guangzhou, Guangdong, China; ^2^Guangdong Research Institute for Orthopedics and Traumatology of Chinese Medicine, Guangzhou, Guangdong, China

**Keywords:** perioperative blood loss, percutaneous endoscopic interlaminar discectomy, unilateral biportal endoscopic, open fenestration discectomy, lumbar disc herniation, hidden blood loss

## Abstract

**Objective:**

The perioperative blood loss of percutaneous endoscopic interlaminar discectomy (PEID), unilateral biportal endoscopic (UBE) and open fenestration discectomy (OPD) was compared to provide reference for the selection of clinical surgical methods.

**Methods:**

The clinical data of 260 patients with lumbar disc herniation who underwent PEID, UBE or OPD surgery from March 2020 to February 2024 were retrospectively analyzed, including 100 patients who received PEID surgery, 100 patients who received UBE surgery, and 60 patients who received OPD surgery. Total blood loss and hidden blood loss were calculated according to the linear equation of circulating blood volume, and the hidden blood loss was statistically compared among the three groups.

**Results:**

In terms of perioperative total blood loss and visible blood loss, the OPD group had the highest total blood loss (408.46 ± 116.89 mL) and visible blood loss (127.17 ± 24.22 mL), followed by the UBE group (304.46 ± 87.55 mL and 51.00 ± 11.15 mL respectively). The PEID group was the least (152.87 ± 54.48 mL and 18.75 ± 5.09 mL). Both the overall differences among the three groups and the pairwise differences were statistically significant (*p* < 0.05). As for hidden blood loss, the results indicated significant differences between the PEID and UBE groups (*p* < 0.05), as well as between the PEID and OPD groups (*p* < 0.05). However, no significant difference was observed between the OPD group and the UBE group (*p* = 0.22). In terms of operation time, UBE group had the longest operation time (129.67 ± 30.56 min), and OPD group had the shortest operation time (78.73 ± 11.80 min), with statistical difference (*p* < 0.05).

**Conclusion:**

In terms of perioperative blood loss, the PEID group was more minimally invasive than the UBE and OPD groups. Compared with OPD group, UBE group was less invasive, but did not significantly reduce the amount of hidden blood loss after surgery. In terms of operation time, UBE group had the longest operation time and OPD group had the shortest operation time. In terms of hospitalization days, OPD group had the longest hospital stay. In terms of total hospitalization cost, UBE group had the highest total hospitalization cost and PEID group had the lowest total hospitalization cost. The clinician should choose the appropriate surgical plan according to the actual situation of the patient to ensure the efficacy and safety of the operation.

## 1 Introduction

With the development of society and the change of lifestyle, the incidence of lumbar disc herniation (LDH) has gradually increased (1). The main symptoms of LDH are low back pain, radiating pain or sensory disturbance of lower limbs, and decreased muscle strength (2). Although non-surgical treatment is still the main treatment method, surgical treatment is often required with the aggravation of the disease or ineffective non-surgical treatment. Open fenestration discectomy (OPD) is a classic surgical procedure for LDH (3). In recent years, with the development of spinal minimally invasive technology and the invention of related instruments, several minimally invasive surgical procedures, such as Percutaneous Endoscopic Interlaminar Discectomy (PEID) and Unilateral Biportal Endoscopic (UBE), have been gradually introduced into clinical practice (4–8). These procedures have been widely adopted in clinical settings and have achieved favorable curative effects. The purpose of minimally invasive surgery is to reduce intraoperative interference with normal tissues and reduce bleeding, so as to promote rapid postoperative recovery (9, 10).

Clinical assessment of intraoperative blood loss is mainly to calculate the intraoperative gauze infiltration and the difference between the amount of fluid in the suction cylinder and the amount of irrigation during the operation. However, because both PEID and UBE are performed in aqueous media, it is difficult to accurately calculate the amount of blood loss associated with surgery. This is due to continuous flushing and blood infiltration into the soft tissues or staying in the dead zone of the surgical channel (11). Sehat et al. (12) believed that invisible bleeding caused by postoperative blood infiltration into muscle space, potential lacunae and hemolysis resulted in this phenomenon, and thus proposed the concept of “hidden blood loss.” This kind of hidden blood loss is a special form of blood loss which is difficult to be directly estimated and easy to be neglected in clinic.

In recent years, many studies have been conducted to compare the application of PEID, UBE, and OPD, as these procedures are performed through the posterior lumbar interlaminar approach to remove the herniated disc. Most of those studies are compared from the aspects of postoperative efficacy and complications (13–15). However, there are few studies on perioperative blood loss. Accurately assessing the amount of hidden blood loss in different surgical methods is conducive to evaluating surgical risks and detecting changes in the patient’s condition. It is also beneficial for reducing perioperative-related complications and lowering the risk of infection. Additionally, it enables the implementation of measures to prevent thrombus formation according to the specific situation, thus ensuring patient safety. Therefore, this study observed and compared the perioperative blood loss of these three different posterior nonfusion decompression operations, in order to provide reference for the selection of clinical surgical methods.

## 2 Patients and methods

### 2.1 Inclusion criteria and exclusion criteria

Inclusion criteria:

1. The patient was clinically diagnosed as LDH, and MRI indicated that L4/5 or L5/S1 single-level unilateral disc herniation compressed the nerve root. The clinical symptoms and signs were consistent with the imaging results, and X-ray of the lumbar spine showed no obvious lumbar spondylolisthesis or severe instability.
2. More than 3 months non-surgical treatment is ineffective or acute prominent symptoms are serious, seriously affecting daily work and life. PEID, UBE or OPD surgery was performed.
3. Blood analysis was performed before surgery and on the second day after surgery.

Exclusion criteria:

1. Symptoms of multilevel disc herniation;
2. Combined with lumbar tumors, tuberculosis, infection, or extensive lumbar spinal stenosis;
3. Have diseases of the blood system or coagulation dysfunction;
4. Perioperative anticoagulant or antiplatelet aggregation drugs were taken;
5. Patients with mental illness or serious medical disease.

### 2.2 Patients and general information

The data of patients with lumbar disc herniation who underwent PEID, UBE or OPD surgery in the Third Affiliated Hospital of Guangzhou University of Chinese Medicine from March 2020 to February 2024 meeting the above criteria were retrospectively analyzed. A total of 260 patients were enrolled, including 100 patients who underwent PEID surgery, 100 patients who underwent UBE surgery, and 60 patients who underwent OPD surgery. The surgical method was mainly determined according to the characteristics of the compressors, and OPD surgery was given priority for the disc tissue with severe protrusion, prolapse or even free into the spinal canal. For mild to moderate disc herniation, PEID or UBE surgery was preferred. The final surgical method was determined by strictly grasping the indications and combining the results of doctor-patient communication. This study was approved by the Hospital Ethics Committee and all patients gave informed consent (No. PJ-KY-20221227-0031).

Clinical data, including patient’s gender, age, height, weight, whether he or she suffers from internal diseases such as hypertension and diabetes, surgical segment, surgical method, preoperative coagulation, preoperative and postoperative blood analysis on the second day, intraoperative blood loss and operation time, total hospitalization days and hospitalization expenses were systematically collected. Among them, preoperative coagulation included prothrombin time (PT), international normalized ratio (PT-INR), activated partial thromboplastin time (APTT), and fibrinogen (FIB). Blood analysis included hemoglobin (Hb), hematocrit (Hct), etc.

### 2.3 Surgical procedures

All patients were given general anesthesia by tracheal intubation or continuous epidural anesthesia, and were placed in prone position on the fluoroscopic operating bed. According to fluoroscopic positioning by C-arm machine, routine disinfection and towel placement were performed.

**Figure 1:**
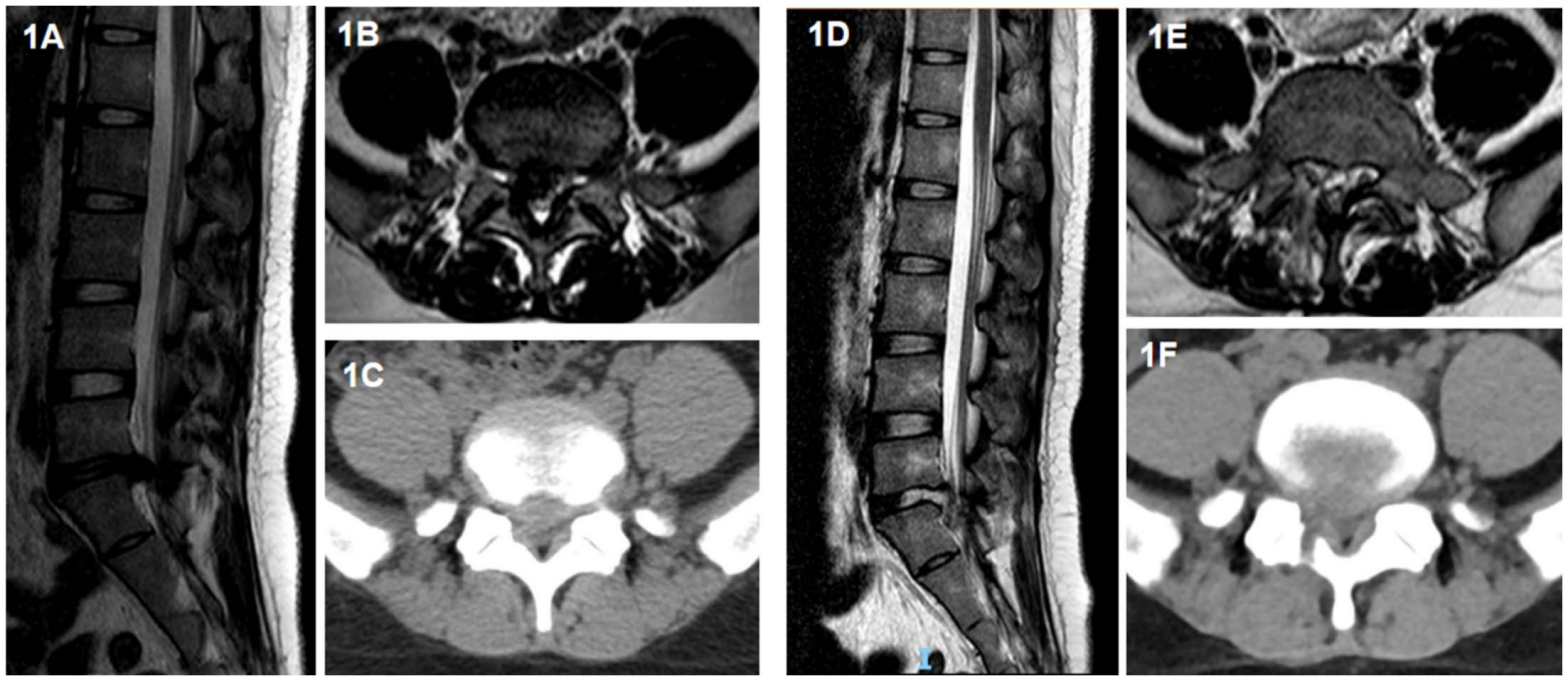
A 35-year-old female patient with right-sided protrusion of the L5/S1 intervertebral disc who underwent PEID surgery. **(A)** Sagittal view of preoperative lumbar magnetic resonance imaging (MRI); **(B)** horizontal view of preoperative lumbar MRI; **(C)** horizontal view of preoperative lumbar computed tomography (CT); **(D)** sagittal view of postoperative lumbar MRI; **(E)** horizontal view of postoperative lumbar MRI; **(F)** horizontal view of postoperative lumbar CT.

PEID surgery: a longitudinal incision of about 8 mm was made at 0.5–1 cm beside the spinous process of the responsible segment. The catheter pencil tip was placed on the upper margin of the laminae of the lower vertebral body, and was progressively expanded with a 3-stage cannula. The depth of cannula was confirmed under fluoroscopy, and the working cannula was placed after the position was satisfied. Then the endoscope was connected, the electrocoagulation knife stopped the bleeding, and the nerve root stripper carefully revealed the nerve root. After the nucleus pulposus protrusion and part of the nucleus pulposus in the intervertebral disc were removed by the nucleus pulposus forceps under the endoscope, another exploration was conducted to confirm that there was no significant residual nucleus pulposus tissue in the spinal canal and the nerve root decompression was complete. Under the endoscope, the dural sac was seen to pulsate with respiration, and the annulus fibrosus was formed using radiofrequency knife. After completion, the examination was conducted again. If there was obvious bleeding, radiofrequency knife was used to stop bleeding under the endoscope. Finally, after the endoscope and working cannula were exited, the suture was performed to complete the operation. Generally, it did not place drainage. If the operation time was longer or the bone structure was worn away more, the drainage was considered as appropriate (Figure 1).

**Figure 2:**
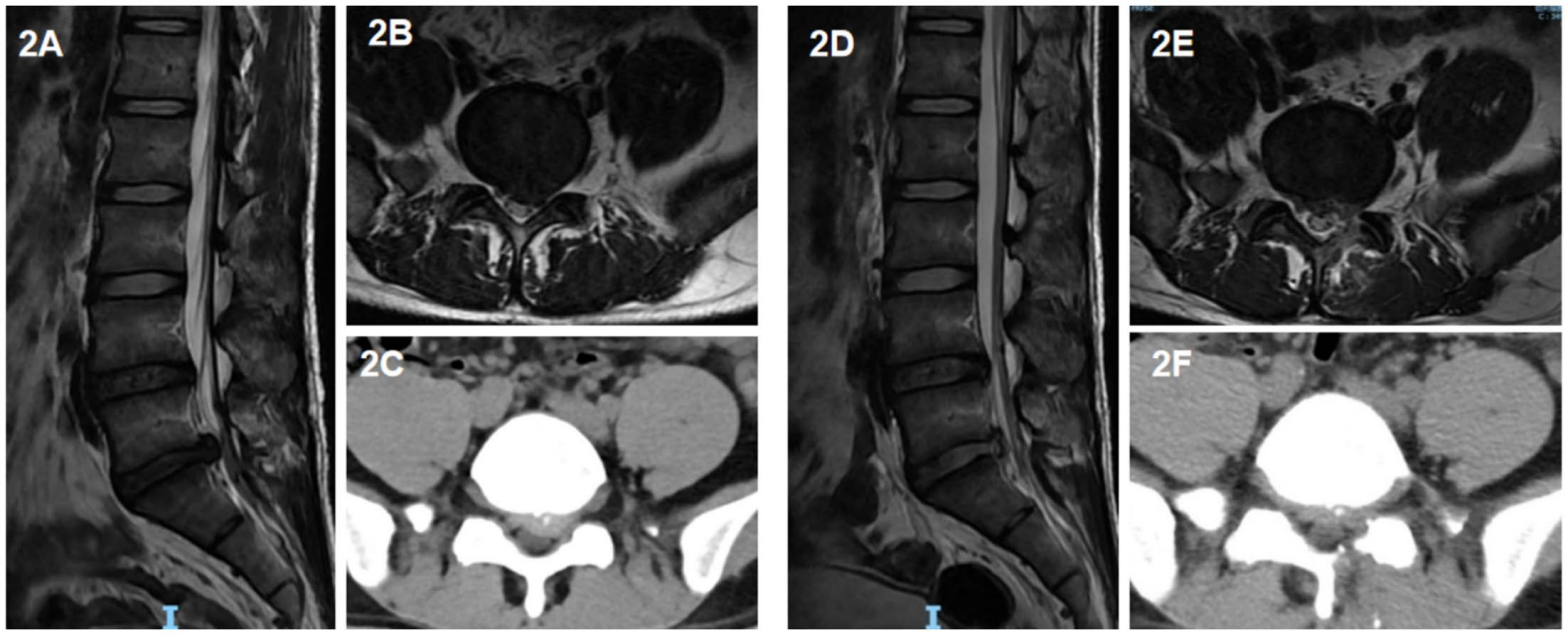
A 39-year-old male patient with left-sided protrusion of the L5/S1 intervertebral disc who underwent UBE surgery. **(A)** Sagittal view of preoperative lumbar MRI; **(B)** horizontal view of preoperative lumbar MRI; **(C)** Horizontal view of preoperative lumbar CT; **(D)** sagittal view of postoperative lumbar MRI; **(E)** horizontal view of postoperative lumbar MRI; **(F)** horizontal view of postoperative lumbar CT.

UBE surgery: the dual channels were located on the medial side of the upper and lower pedicle lines. A horizontal transverse line was made for the intervertebral space of the responsible segment. The horizontal transverse line and the intersection point of the medial pedicle line 15 mm at the head end and 15 mm at the tail end were double channels. One channel is the endoscopic observation channel, and the other side is the operation channel. The positions of the two channels could be changed according to the situation. The skin and deep fascia were incised at the double channel, and the cannula was placed at the junction of the spinous process and laminae. Soft tissue dilation and muscle dissection were performed, endoscope and working channel were inserted, respectively. Then the endoscope was connected, the electrocoagulation knife stopped the bleeding, and the nerve root stripper carefully revealed the nerve root. After the nucleus pulposus protrusion and part of the nucleus pulposus in the intervertebral disc were removed by the nucleus pulposus forceps under the endoscope, another exploration was conducted to confirm that there was no significant residual nucleus pulposus tissue in the spinal canal and the nerve root decompression was complete. Under the endoscope, the dural sac was seen to pulsate with respiration, and the annulus fibrosus was formed using radiofrequency knife. After completion, the examination was conducted again. If there was obvious bleeding, radiofrequency knife was used to stop bleeding under the endoscope. Finally, the endoscope and working channel were exited, and the suture orifice after the negative pressure drainage tube was indwelled to end the operation (Figure 2).

OPD surgery: with the intervertebral space of the responsible segment as the center, a longitudinal incision of 3–5 cm in length was made through the posterior median approach. The paravertebral muscle was dissected layer by layer along the subperiosteum to reveal the laminae and articular process of the responsible segment. The laminae were fenestrated to reveal and remove the protruding nucleus pulposus. The spinal canal and nerve root canal were examined again to confirm that the nerve root had been loosened. Finally, the operation was completed by full hemostasis, irrigation, indwelling negative pressure drainage tube and suturing layer by layer.

### 2.4 Postoperative management

The patient received prophylactic antibiotics within 24 h after surgery, combined with mannitol, dexamethasone, nutrophin and other drugs. The patient rested in bed for 24 h, and raised the straight leg of the affected lower limb in bed to prevent nerve root adhesion. Those with drainage tubes were removed after the operation with a drainage volume <50 mL. 24 h after surgery, the patient could gradually sit and walk after wearing the waist, and gradually exercise the lumbar muscle.

### 2.5 Calculation of blood loss

Preoperative blood volume (PBV), perioperative total blood loss (TBL), and hidden blood loss of patients were calculated by reviewing patient case data. PBV was calculated using Nadler’s formula (16):

$$\mathrm{PBV}=k_{1}\times {\text{Height}}^{3}(m)+k_{2}\times \text{Weight}(\mathrm{kg})+k_{3}$$

For males: k_1_ = 0.3669, k_2_ = 0.03219, k_3_ = 0.6041; For females: k_1_ = 0.3561, k_2_ = 0.03308, k_3_ = 0.1833.

The perioperative human circulation blood volume equation proposed by Gross (17) was used to calculate the perioperative TBL:

$$\begin{array}{l} \mathrm{TBL}=\mathrm{PBV}\times ({\mathrm{Hct}}_{\text{post}}-{\mathrm{Hct}}_{\mathrm{pre}})/{\mathrm{Hct}}_{\mathrm{ave}}\;\text{and}\;\\ {\mathrm{Hct}}_{\mathrm{ave}}=({\mathrm{Hct}}_{\text{post}}+{\mathrm{Hct}}_{\mathrm{pre}})/2 \end{array}$$

At the same time, TBL = visible blood loss+ hidden blood loss - transfusion volume. The visible blood loss included intraoperative blood loss and postoperative drainage volume. Since there was no transfusion in the perioperative period,

hidden blood loss=TBL−visible blood loss

### 2.6 Statistical analysis

SPSS 25.0 software (IBM Corp., Armonk, NY, United States) was used for statistical analysis, and the measurement data were described as mean ± standard deviation. Counting data was described numerically, representing the number of cases. The Kruskal-Wallis test was employed for the global comparative analysis of the three groups of data. For indicators such as visible blood loss, hidden blood loss, total blood loss, and tospital stay, the Wilcoxon-Mann–Whitney test was then used for pairwise comparisons. The Chi-square test was applied to the count data, and *p* < 0.05 was considered statistically significant.

## 3 Results

A total of 260 patients were included in this study, including 100 patients who received PEID surgery, 100 patients who received UBE surgery, and 60 patients who received OPD surgery. The general information of the three groups of patients were shown in Table 1. All patients successfully completed the operation without serious complications such as injury of important blood vessels or nerves. There were no significant differences in age, height, weight, gender, segmental distribution and internal diseases among the three groups (*p* > 0.05).

**Table 1:** The general information of the patients.

Characteristic	PEID group (*n* = 100)	UBE group (*n* = 100)	OPD group (*n* = 60)	*P*-value
Age (years)	51.00 ± 14.66	52.21 ± 14.21	52.45 ± 16.35	0.83
Height (cm)	165.84 ± 5.73	166.22 ± 5.81	165.77 ± 6.40	0.84
Body weight (kg)	59.42 ± 8.68	61.43 ± 10.83	60.05 ± 11.00	0.41
Gender (*n*)				
Male	58.00	59.00	31.00	0.64
Female	42.00	41.00	29.00	
Disc level (*n*)				
L4/5	31.00	39.00	27.00	0.19
L5/S1	69.00	61.00	33.00	
Hypertension (*n*)	28.00	23.00	19.00	0.47
Diabetes (*n*)	13.00	17.00	9.00	0.73

The *p-*value represents the overall results of the three group comparison analyzed by the Kruskal-Wallis test or the Chi-square test.

There were no statistically significant differences in preoperative coagulation function indexes (PT, PT-INR, APTT, FIB), Hb and Hct among the three groups of patients (*p* > 0.05), as shown in Table 2. However, there were statistically significant differences in postoperative Hb and Hct among the three groups (*p* < 0.05). Among them, the OPD group had the most significant decline. In addition, the operation time, hospitalization days and total hospitalization cost of the three groups were compared, and the differences were statistically significant (*p* < 0.05). In terms of operation time, UBE group had the longest operation time and OPD group had the shortest operation time. In terms of hospitalization days, when compared with either the PEID group or the UBE group respectively, OPD group had the longest hospital stay (*p* < 0.05). However, when comparing the hospital stays between the PEID Group and the UBE Group, there was no significant difference (*p* = 0.48). In terms of total hospitalization cost, UBE group had the highest total hospitalization cost and PEID group had the lowest total hospitalization cost.

**Table 2:** Comparison of perioperative data of PEID, UBE, and OPD Group.

Outcomes	PEID group (*n* = 100)	UBE group (*n* = 100)	OPD group (*n* = 60)	*P*-value
Pre-op coagulation function				
PT	10.87 ± 1.35	11.09 ± 1.34	11.07 ± 1.24	0.54
PT - INR	0.94 ± 0.10	0.95 ± 0.10	0.92 ± 0.07	0.32
APTT	23.98 ± 3.19	24.65 ± 3.41	24.29 ± 3.30	0.49
FIB	2.84 ± 0.61	2.96 ± 059	2.92 ± 0.49	0.36
Pre-op Hb (g/L)	127.89 ± 15.63	127.22 ± 13.01	125.87 ± 15.16	0.74
Post-op Hb (g/L)	123.28 ± 15.74	116.43 ± 12.95	110.57 ± 15.53	<0.01
Pre-op Hct (%)	40.30 ± 2.00	40.29 ± 2.19	40.53 ± 1.88	0.62
Post-op Hct (%)	38.14 ± 1.98	37.41 ± 2.06	36.62 ± 1.79	<0.01
Visible blood loss (mL)	18.75 ± 5.09	51.00 ± 11.15	127.17 ± 24.22	<0.01
Total blood loss (mL)	152.87 ± 54.48	304.46 ± 87.55	408.46 ± 116.89	<0.01
Hidden blood loss (mL)	134.12 ± 53.94	253.46 ± 88.52	281.29 ± 115.31	<0.01
Operation time (min)	116.72 ± 29.37	129.67 ± 30.56	78.73 ± 11.80	<0.01
Hospital stay (days)	6.80 ± 1.24	6.69 ± 1.20	8.28 ± 1.26	<0.01
Total hospitalization costs (¥)	18363.35 ± 1914.74	23142.42 ± 1375.05	19482.61 ± 2434.07	<0.01

The *P-*value represents the overall results of the three group comparison analyzed by the Kruskal-Wallis test.

**Figure 3:**
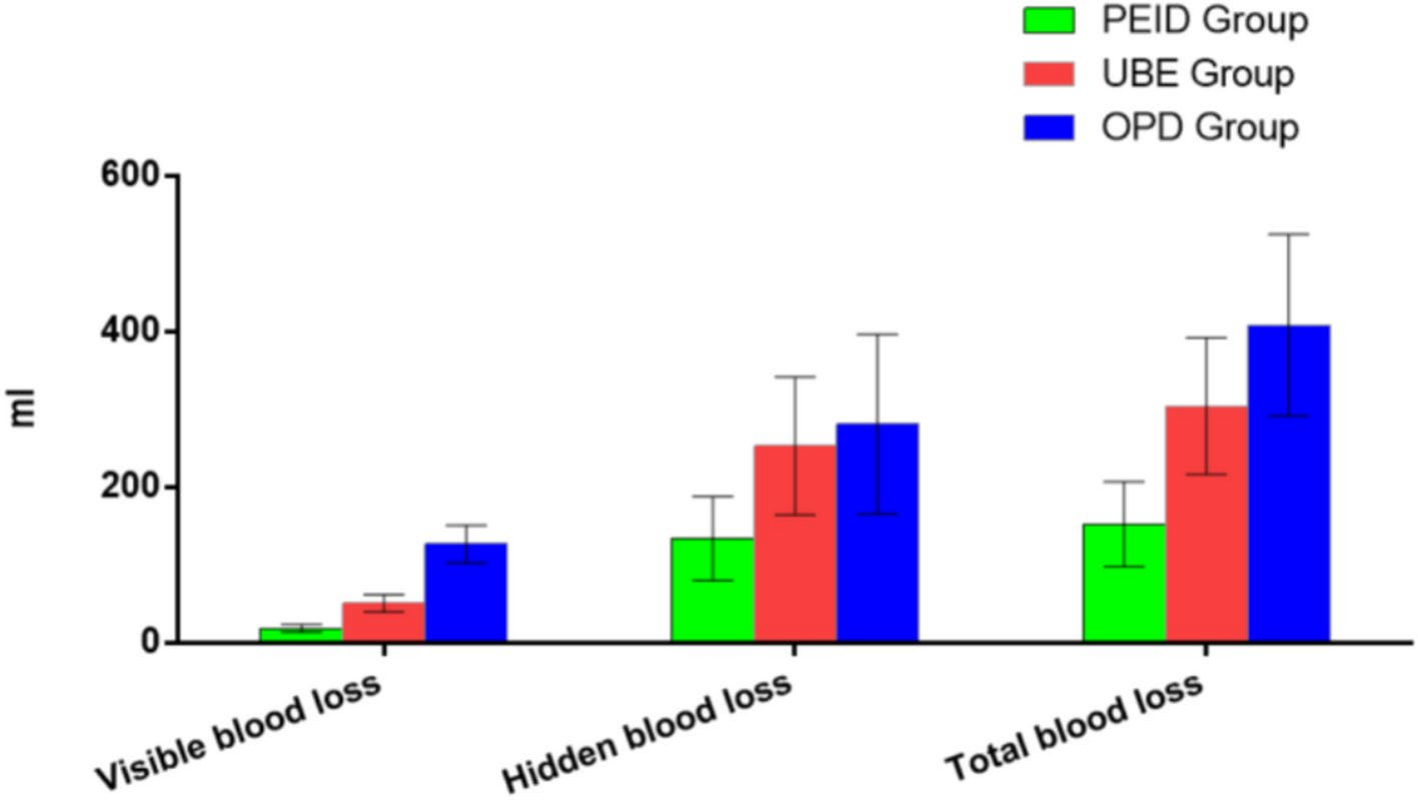
Comparison of different types of blood loss among the PEID, UBE, and OPD Group.

Perioperative blood loss analysis of the three groups showed that in terms of total blood loss and visible blood loss, the OPD group had the highest total blood loss (408.46 ± 116.89 mL) and visible blood loss (127.17 ± 24.22 mL). The UBE group (304.46 ± 87.55 mL and 51.00 ± 11.15 mL, respectively) was followed by the PEID group (152.87 ± 54.48 mL and 18.75 ± 5.09 mL). Both the overall differences among the three groups and the pairwise differences were statistically significant (*p* < 0.05). In terms of hidden blood loss, the Kruskal-Wallis test showed statistically significant overall differences among the PEID, UBE, and OPD groups (*p* < 0.05). Subsequently, pairwise comparisons were performed using the Wilcoxon-Mann–Whitney test. The results indicated significant differences between the PEID and UBE groups (*p* < 0.05), as well as between the PEID and OPD groups (*p* < 0.05). However, no significant difference was observed between the OPD group and the UBE group (*p* = 0.22), as shown in Figure 3.

## 4 Discussion

For a long time, spinal surgeons mainly paid attention to intraoperative blood loss and postoperative drainage volume, and paid insufficient attention to the hidden blood loss after surgery. Previous studies have found that hidden blood loss was much larger than visible blood loss (13, 18), which is consistent with the results of this study. Although the exact cause of hidden blood loss was not yet clear, there was still a significant link between the trauma of the surgery and the amount of blood lost (19, 20). In addition, it was also related to potential lacunae formed during surgery (21), blood extravasation into tissue space and destruction of red blood cell due to hemolysis (22). Some scholars have also suggested that factors such as long operation time, multiple surgical segments, the use of tranatemic acid, and postoperative free fatty acids in blood circulation may affect hidden blood loss (23, 24). What is more, the patient’s own factors were also important factors for hidden blood loss, such as age and their own underlying diseases, coagulation disorders and so on. Excessive blood loss could lead to longer wound healing time, increased risk of infection, increased bed time and increased risk of related complications (25). Therefore, more attention should be paid to reducing blood loss in clinical work, which is very important to promote postoperative rehabilitation of patients.

In this study, it was found that OPD group had the highest total blood loss and visible blood loss, followed by UBE group and PEID group at least. However, there was no significant difference between the OPD group and the UBE group in hidden blood loss, while the PEID group was significantly less. As a traditional surgical method for disc herniation, OPD surgery required paravertebral muscle dissection, muscle stretching, and fenestration in the lamina, theoretically resulted in greater injury and more blood loss than UBE and PEID channel surgery. The paravertebral muscle surrounding the target segment required to be stripped and cleared during UBE luminoplasty to obtain adequate visibility, which may cause more damage to soft tissue than single-channel PEID surgery to expose the laminar space. On the other hand, artificially created potential lacunae provided space for the generation and inflow of hidden blood loss after surgery. Larger potential lacunae may lead to increased hidden blood loss, and this lacunae cannot be closed and reduced by suture after surgery. UBE surgery was a dual channel and PEID surgery was a single channel, so it was clear that the potential lacunae created by UBE surgery were larger than those created by PEID surgery. Interestingly, we found no significant difference in hidden blood loss between the OPD and UBE groups. This suggested that there seemed to be no significant difference in the potential damage caused by UBE and OPD surgery. This may be related to the following factors. First of all, compared to OPD surgery, UBE surgery also requires removal of lumbar laminae and disc, and their general surgical procedures are similar to the affected tissue structure. UBE surgery has less tissue damage, which has been reflected in the visible blood loss. Secondly, the operative time of UBE surgery is longer, and the operative time is an important factor affecting the amount of latent blood loss (26). In addition, OPD surgery is an open operation, which is conducive to electrocoagulation or pressure hemostasis during the operation, and hemostatic materials can be directly applied if necessary. However, the operating range and visual field of UBE surgery are relatively limited, resulting in poor intraoperative hemostatic effect and increased hidden blood loss after surgery.

Clinicians should make comprehensive consideration when selecting the surgical plan according to the actual situation. Although spinal endoscopy can reduce surgical trauma caused by paravertebral soft tissue dissection, other considerations are also necessary, such as effective decompression (27). Compared with the other two kinds of endoscopic surgery, OPD surgery has relatively larger trauma and more blood loss, which may destroy part of the structure of the spine and affect the stability of the spine. However, it has a wide range of indications, and has good therapeutic effect for various types of lumbar disc herniation, especially complex cases. In addition, it can fully expose the surgical area, making decompression more thorough, operation more convenient, and technology more mature (28). PEID surgery is less invasive, less bleeding, and has less impact on the stability of the spine. However, this technology requires high requirements and a long learning curve, which requires doctors to have certain operating skills and experience. In addition, its indications are relatively narrow, for some complex lumbar disc herniation, it may not be fully applicable. Moreover, due to the limited visual field and operating space of PEID surgery, there may be incomplete decompression (29). UBE surgery has two channels, and its field of view is relatively clearer and the operation is more flexible. The size of the incision and the damage to the paravertebral muscle are between the previous two surgeries. However, its disadvantage is that its equipment requirements and surgical costs are relatively high. Moreover, due to the relative complexity of the operation, the operation time is often longer than that of traditional open surgery (30). In addition, there are also steep learning curves and relatively high requirements for doctors.

Although the above research results have certain clinical significance, there are some limitations. First of all, this is a retrospective study, and there may be selection bias in the included cases. Second, the assessment of apparent blood loss may be slightly inaccurate for the effect of intraoperative irrigation. In addition, although some studies have suggested that the hemodynamics of patients on the second or third day after surgery have been nearly stable, and liquid transfer has been basically completed (31). But if patients continue to lose blood, it may affect the reliability of the results. Furthermore, due to the absence of subsequent follow-up records for some patients in this study, the subsequent follow-up data were not incorporated. Nevertheless, this has no impact on the evaluation outcomes of the perioperative period. Therefore, further studies need to evaluate the multiple results of blood analysis and the data from subsequent follow-ups, or prospective, large-cohort studies should be conducted for further confirmation.

## 5 Conclusion

In summary, PEID is significantly superior to UBE and OPD in terms of overall blood loss. Although the visible blood loss of UBE was significantly less than that of OPD, there was no significant difference in the hidden blood loss. In terms of operation time, UBE group had the longest operation time and OPD group had the shortest operation time. In terms of hospitalization days, OPD group had the longest hospital stay. In terms of total hospitalization cost, UBE group had the highest total hospitalization cost and PEID group had the lowest total hospitalization cost. In clinical practice, PEID can be given priority in the treatment of lumbar disc herniation on the premise of ensuring safety and sufficient decompression. The clinician should understand the advantages and disadvantages of various surgical methods and choose the appropriate surgical plan according to the actual situation of patients to ensure the efficacy and safety of surgery.

## Data Availability

The original contributions presented in the study are included in the article/supplementary material, further inquiries can be directed to the corresponding author.
